# Effects of Recovery-Orientation on the Use of Forced Medication and Maximum Daily Drug Dose: The “Weddinger Modell”

**DOI:** 10.3389/fpsyt.2021.789822

**Published:** 2021-12-15

**Authors:** Klara Czernin, Felix Bermpohl, Alexandre Wullschleger, Lieselotte Mahler

**Affiliations:** ^1^Department of Psychiatry, St. Hedwig Hospital, Charité University Medicine Berlin, Berlin, Germany; ^2^Department of Child and Adolescent Psychiatry, Medical University of Vienna, Vienna, Austria; ^3^Division of Adult Psychiatry, Department of Psychiatry, Geneva University Hospitals, Thonex, Switzerland; ^4^Department of Psychiatry, Clinics in the Theodor-Wenzel-Werk, Berlin, Germany

**Keywords:** coercion, forced medication, acute psychiatry, Recovery, Weddinger Modell

## Abstract

**Objective:** The aim of the present study was to analyze the effects of the implementation of the Recovery-orientated psychiatric care concept “Weddinger Modell” on the incidence of forced medication, the total number of forced medication incidents per affected case, the maximum dose of a singular forced medication and the maximum voluntary daily drug dose of different psychotropic drugs administered during an inpatient stay.

**Methods:** This retrospective case-control study included 234 patients. A pre/post-comparison of patients on two acute psychiatric wards before (control group, *n* = 112) and after (intervention group, *n* = 122) the implementation of the Weddinger Modell in 2010 was performed. Patient data was selected at two reporting periods before and at two reporting periods after 2010.

**Results:** No significant differences were found in the incidence of forced medication and the total number of forced medications. A significant reduction of the maximum forced medication dose of haloperidol in the intervention group was seen. Furthermore, the analysis of the intervention group showed a significant reduction of the maximum voluntary daily drug doses of clozapine, haloperidol and risperidone.

**Discussion:** The results indicate that the implementation of the Weddinger Modell had no effect on the incidence of forced medication, but it can help to improve the approach to psychotropic drugs. Despite the reduction of mechanical coercive measures by the model, as shown in a previous study, there is no increase in forced medications or administered drug doses. Focus on Recovery helps in reducing coercion in acute psychiatric care.

## Introduction

Coercive measures, such as seclusion, restraint, and forced medication have been used since the beginning of psychiatric treatment to avoid acute endangerment of the patient or others. They should only be used as a last resort when all therapeutic options have been exhausted (1). Nevertheless, coercive measures are considered to pose a grave infringement of fundamental and basic human rights (2). Therefore, clinicians are confronted with a difficult ethical dilemma between the use of coercion due to urgent need for intervention to prevent danger and the loss of patient's autonomy. Applications of coercive measures can be connected to significant negative consequences for affected patients, even the development of trauma-related symptoms has been described (3). Although progress has been made within the psychiatric system to reduce the use of coercive measures, incidences of coercive measures are still numerous (4) and their prevention has become the subject of scientific discussions, research projects and treatment initiatives (5–7).

Forced medication is considered the least acceptable and at the same time most frequently used coercive intervention (56%) in mental health practice in Europe, followed by restraint (36%) and seclusion (8%) (4, 8). Forced medication is defined either as the application of parenteral medication by force or oral medication under the threat of forced parenteral medication if oral intake is refused (9). Forced medication has an important impact on patient disapproval of involuntary treatment (10). Possible predictive factors for the use of forced medication are some patients' characteristics such as male gender, younger age, a psychotic or mood disorder, homelessness, substance abuse, and migration background (11, 12). Also, staff attitudes and ward characteristics play a role in the application of forced medication (13). Recommendations for good clinical practice on forced medication include that forced medication can only be used if a therapeutic intervention is urgently needed, the voluntary intake of medication is consistently rejected and the patient is not capable of giving consent (5).

In order to change long-term therapeutic processes and to improve acute psychiatric care a novel treatment concept, the Weddinger Modell, was implemented in December 2010 at the Psychiatric University Clinic of the Charité at the St. Hedwig Krankenhaus (PUK SHK) in Berlin (14). The model is based on the theoretical concepts of recovery, salutogenesis, and empowerment. It promotes participation and transparency as well as a patient-centered and needs-based treatment which requires individualization of the therapy. Treatment goals and individual treatment elements are continuously evaluated by the patient and the multi-professional team. The focus of this approach is on the resources, strategies, experiences as well as the patient's illness and recovery concepts. The treatment setting is flexible (in-patient, partly in-patient as well as out-patient) without impacting the continuity of therapeutic relationship. A further core element is the multidisciplinary design of the treatment: The closely cooperating multi-professional primary therapist teams share responsibility for treatment and the relevance of the different professional groups is adapted to the individual requirements of the respective patients. Additionally, the active inclusion of the patient's caregiver networks (including network meetings, rooming-in, and relative consultations) as well as an open-door policy, and the extension of the team by experts through experience (peer-consultation) are counted as further essential elements (15). The Weddinger Modell requires changes in traditional ward structures and conventional treatment processes. This means that team meetings and treatment planning efforts are held in the presence of the patients themselves. Thereby, the patients can actively participate in the planning of the interventions and adjust the treatment procedure.

The reduction of coercive measures is a declared goal of the Weddinger Modell. Essential for this approach is the transparent and multidisciplinary decision-making process as well as an obligatory post-coercion review session of any coercive measure, which was further standardized and investigated in 2017 (16). A recent study has shown that the review sessions can reduce negative consequences of coercive interventions such as symptoms of post-traumatic stress disorder (17). The focus on participation and active treatment modeling by the patients correlate to a decrease in the subjective experience of coercion (18). The careful use of psychotropic drugs is another main goal of the model, especially the treatment with antipsychotics in minimum effective dosage and the focus on non-pharmacological interventions.

A previous study in this population showed that the Weddinger Modell led to a reduction of mechanical coercive measures (19). Due to the previously described negative correlation between forced medication and mechanical coercive measures, it might be possible that mechanical coercive measures were reduced in favor of an increase of forced medication and higher doses of psychotropic drugs (20, 21). However, until now, the effects of the model on forced medication and maximum daily drug doses (DDDs) have not been systematically investigated. The aim of the present study was to analyze whether the implementation of the Weddinger Modell on two acute psychiatric wards does lead to a measurable change in forced medication and the maximum DDD.

## Materials and Methods

We performed a retrospective case control study by individually screening archived original patient files and electronic documentation. This study received a positive vote from the Ethics Committee of the Charité (reference number: EA2/106/18).

Recruitment of patients took place at the two general psychiatric wards 36 and 37 at PUK SHK. Each ward is responsible for a defined geographical catchment area. The two wards resemble each other in architecture, size (maximum capacity of 30 beds), staff, catchment area region characteristics and their focus on the treatment of severe and acute mental illness. The Weddinger Modell was established in December 2010. There were no major differences between the wards at the time of the survey. According to the Sozialstrukturatlas Berlin 2013 there were comparable conditions in their catchment areas with regard to low socio-structural factors and an unfavorable assessment of social and health burden (22). In order to reduce other influences which could have caused bias, such as the publication of new guidelines, the ratification of the United Nations convention on the rights of disabled people in 2009, we chose multiple time points and adapted our study to a pre/post-design.

We chose four observation periods of equal length (48 h) mid-week (Wednesday and Thursday) in a similar seasonal period (November) of the years 2005 (November 16–17), 2009 (November 18–19), 2011 (November 16–17), and 2013 (November 13–14). The selection of observation periods was guided by the fact that later changes to the regional jurisdiction concerning coercive measures in treatment of psychiatric disorders (Berlin PsychKG of 2016) were not effective then. All patients who were treated in the defined periods were included into the study, hence adding up to a total study population of *n* = 234. The intervention group comprised *n* = 122 patients treated on wards 36 and 37 at the time of data collection in 2011 and 2013. Incidence of involuntary (forced) medication and maximum DDD of psychotropic drugs over the entire inpatient stay was individually assessed.

### Definitions

Forced medication describes the extraordinary administration (parenteral or oral) of psychotropic drugs by force or against the will of the patient. The legal regulations for coercive measures including forced medication in psychiatric facilities in Berlin are established by a collection of federal and local legislations such as PsychKG, BGB and StGB (justifying emergency).

Benzodiazepines were converted to an equivalent dose of diazepam using a conversion scheme. 0.5 mg clonazepam, 1 mg lorazepam, 1 mg lormetazepam, 20 mg oxazepam, and 50 mg tetrazepam were converted to 10 mg diazepam.

### Outcome Parameters

#### Incidence Forced Medication

“Incidence forced medication” describes how many patients were affected by forced medication during their inpatient stay. It is given as a total number *n* and percent of the respective study group.

#### Events of Forced Medication per Affected Patient

“Events of forced medication per affected patient” describes the total number of forced medication incidents throughout the entire stay per affected case as a median and inter-quartile range (IQR).

#### Maximum Forced Medication Dose

“Maximum forced medication dose” describes the maximum administered singular dose of different psychotropic drugs in a coercive context in mg as a median and IQR.

#### Maximum Daily Drug Dose

“Maximum daily drug dose” describes the maximum voluntarily administered daily dosage of each psychotropic drug throughout the patients' stay in mg as a median and IQR.

#### Length of Stay

“Length of stay” describes the length of stay in hospital in days as a mean and standard deviation (SD).

#### Data Collection

The forced medications and psychotropic drug doses were recorded on the basis of the patient chart, the restraint and seclusion order forms as well as the electronic documentation. Parameters under question were extracted and inserted under pseudonymized patient IDs into an SPSS file.

#### Sociodemographic Data

We collected information on gender, age, history of migration (which was defined as being born outside of Germany) and main ICD-10 diagnosis from the electronic documentation system. Individuals were allocated to clinically relevant diagnostic groups according to the ICD-10 manual: (I) substance use disorders (F1); (II) (non-)affective psychotic disorders (F2, F30, and F31); (III) major depressive disorders and neurotic, stress-related, somatoform disorders (F32, F33, and F4); (IV) personality disorders (F6); (V) rest category (F0, F5, F7, and F9).

#### Medication

Incidence and total number of forced medications were collected for all patients receiving in-patient treatment at the selected time points including the administered substances. Maximum forced administered dose of equivalent dose diazepam, haloperidol and zuclopenthixol acetate was collected.

The maximum DDD of commonly administered psychotropic drugs (equivalent dose diazepam, aripiprazole, amisulpride, clozapine, haloperidol, olanzapine, risperidone, and quetiapine) during inpatient stay was retrieved from the patients' medical files.

#### Statistical Analysis

We performed a pre/post-comparison of patients treated on wards 36/37 before and after the implementation of the Weddinger Modell in 2010. In order to test for group differences in predictive sociodemographic variables we performed Chi squared tests for distribution of gender, diagnosis group, migration background, and nominally scaled variables [incidence forced medication (yes/no)]. Differences in age and ordinally scaled variables (events of forced medication, maximum forced medication dose, and maximum DDD) were assessed using Mann-Whitney *U*-test, and reported as median and IQR. Level of significance was set at *p* < 0.05, and *p* < 0.001 considered as highly significant. Statistical analysis was performed with the statistics program IBM Statistics SPSS Version 21 Apple Macintosh OSX.

## Results

### Sociodemographic Data

The examined study groups did not differ significantly with regard to gender, age, history of migration, and distribution of diagnoses (presented in Table 1).

**Table 1:** Gender, age, history of migration, and distribution of diagnoses (*n* = 234).

	**2005 and 2009**	**2011 and 2013**
	**36 + 37 (control group) (*n* = 112)**	**36 + 37 (intervention group) (*n* = 122)**
Men, *n* (%)	63 (56.3%)	60 (49.2%)
Age, mean (SD)	39 (15)	43 (14)
History of migration, *n* (%)	43 (40.2%)	34 (29.1%)
Distribution of diagnoses 1/2/3/4/5, *n*	6/70/25/9/2	3/80/32/4/3
Substance use disorders (F1), *n* (%)	6 (5.4%)	3 (2.5%)
(Non-)affective psychotic disorders (F2, F30, F31), *n* (%)	70 (62.5%)	80 (65.6%)
Major depressive disorders and neurotic, stress-related, somatoform disorders (F32, F33, F4), *n* (%)	25 (22.3%)	32 (26.2%)
Personality disorders, *n* (%)	9 (8.0%)	4 (3.3%)
Rest category (F0, F5, F7, F9), *n* (%)	2 (1.8%)	3 (2.5%)

### Medication

The results are summarized in Table 2.

**Table 2:** Group comparison of psychopharmacologic items and characteristics of coercion before and after implementation of the model (*n* = 234) (*p < 0.05 and **p < 0.001).

	**2005 and 2009**	**2011 and 2013**
	**36 + 37 (control group) (*n* = 112)**	**36 + 37 (intervention group) (*n* = 122)**
Incidence forced medication, *n* (%)	24 (21.43%)	21 (17.21%)
Events of forced medication per affected patient, median (IQR)	2.00 (1.00–6.00)	1.00 (1.00–2.50)
**Maximum forced medication dose**		
Equivalent dose diazepam in mg, median (IQR)	10.00 (10.00–10.00)	10.00 (10.00–10.00)
Haloperidol in mg, median (IQR)	10.00 (10.00–10.00)	5.00 (2.50–10.00)**
Zuclopenthixol acetate in mg, median (IQR)	0.00 (0.00–0.00)	0.00 (0.00–0.00)
**Maximum daily drug dose**		
Equivalent dose diazepam in mg, median (IQR)	30.00 (15.00–35.00) (*n* = 55)	20.00 (10.00–30.00) (*n* = 48)
Aripiprazole in mg, median (IQR)	15.00 (6.26–18.75) (*n* = 12)	20.00 (10.00–30.00) (*n* = 9)
Amisulpride in mg, median (IQR)	800.00 (425.00–800.00) (*n* = 12)	600.00 (400.00–975.00) (*n* = 16)
Clozapine in mg, median (IQR)	600.00 (387.50–700.00) (*n* = 18)	237.50 (87.50–318.75)** (*n* = 20)
Haloperidol in mg, median (IQR)	10.00 (10.00–20.00) (*n* = 31)	10.00 (5.00–15.00)* (*n* = 16)
Olanzapine in mg, median (IQR)	20.00 (13.75–30.00) (*n* = 34)	20.00 (15.00–20.00) (*n* = 41)
Risperidone in mg, median (IQR)	6.00 (4.00–7.00) (*n* = 44)	4.00 (2.38–4.25)** (*n* = 34)
Quetiapine in mg, median (IQR)	500.00 (150.00–1112.50) (*n* = 32)	350.00 (200.00–850.00) (*n* = 28)

### Incidence Forced Medication

In the control group *n* = 24 (21.43%) were affected by forced medication compared to *n* = 21 (17.21%) in the intervention group. The difference was not statistically significant (*X*^2^ = 0.668; *p* = 0.414; *df* = 1).

### Events of Forced Medication per Affected Patient

The intervention group reported a median and IQR for events of forced medication of 1.00 (1.00–2.50), while the control group reported 2.00 (1.00–6.00). The difference was not statistically significant.

### Maximum Forced Medication Dose

The pre/post-intervention comparison showed a significantly lower maximum forced medication dose of haloperidol in the intervention group (*U* = 121.50; *p* = 0.001). The maximum forced medication doses of other psychotropic agents did not differ significantly.

### Maximum Daily Drug Dose

Maximum DDDs of clozapine (*U* = 59.00; *p* < 0.001), haloperidol (*U* = 158.50; *p* = 0.041), and risperidone (*U* = 374.00; *p* < 0.001) were significantly lower for the intervention group when compared to the control group. Other drugs did not differ significantly in their maximum DDDs.

### Length of Stay

Mean lengths of stay did not differ significantly between the study groups [60.1 (*SD* = 51.2) days in the control group vs. 58.6 (*SD* = 51.5) days in the intervention group].

## Discussion

This is the first study to examine the effect of the Weddinger Modell on forced medication and maximum DDDs of administered psychotropic drugs during an in-patient stay. The study contributes to the scarce data on avoidance of coercion in connection with Recovery-oriented and patient-centered therapy approaches. Initial results, indicating that the implementation of the Weddinger Modell was associated with a decrease in mechanical coercive measures, were already published (19).

In the present study no significant differences were found in the incidence of forced medication and the total number of forced medications. Previous research has indicated a negative correlation between the frequency of forced medication and the duration of seclusions, as shown e.g., in the Netherlands (21). This effect was further confirmed by a study on the interim ban of involuntary medication in the state of Baden-Wuerttemberg that had a negative impact on seclusion incidents (20). Although the Weddinger Modell has proven to be effective in reducing the maximum frequency of restraint events as well as the duration of seclusion incidents (19), there was no increase of forced medications in the present study, even though it was part of the same study population. There were even fewer incidents of forced medications recorded, although the difference did not reach statistical significance.

Furthermore, the results showed lower dosages in the intervention group in respect to maximum forced medication dosage of haloperidol and lower maximum DDDs of clozapine, haloperidol, and risperidone. As neuroleptics have significant and potentially severe side effects (23, 24), the lower maximum medication dose administered in the intervention group of patients treated according to the Weddinger Modell should be considered positive and highly relevant. To our knowledge there was no change in prescribing practice regarding combinations of different antipsychotics during that time, which would have had an effect on these parameters. All these results underline the fact that the reduction of mechanical coercive measures observed after the implementation of the Weddinger Modell did not go along with increased dosages of psychotropic drugs during in-patient stay.

Following central aspects of the Weddinger Modell may contribute to the present results:

The cautious and rather reserved handling of psychotropic drugs as part of the model should be seen as an important factor. The use of psychotropic drugs in minimally effective doses and the early focus on other non-pharmacological interventions are part of the handling. This principle also corresponds to the patient's desire for holistic treatment, including psychotherapy and a lower focus on medication (19, 25). The treatment decision for drug therapy is only made participatory and maximally transparent taking into account the effects and side effects. Furthermore, it is already known that incidents of forced medication can disrupt the therapeutic relationship (26), which is seen as the central aspect in the treatment. Therefore, where possible, treatment agreements and crisis plans are drawn up in order to always make decisions oriented as far as possible to the patient's will, even in dangerous situations. Medication against the patient's will is only ultima ratio, as it poses a grave infringement on fundamental and basic human rights and liberties (2).

The special handling of and attitude toward aggression as part of the Weddinger Modell is one of its core elements. Due to the sensitization of the ward teams to treatment against the patient's will, avoidance and reduction of coercion became a basic approach and stated goal of the model. Standardized post-coercion review sessions are considered as an important factor, high importance is attached to evaluate every crisis situation. The therapeutic relationship can be strengthened by the joint review session, in which the respective experience of the acute situation is reflected together. The processes and reasons for the coercive measure are also explained by the team member and made comprehensible for the patient. It can be determined, as shown in a previous study related to mechanical coercive measures (19), that the increased awareness of direct and informal coercion contributes to the present results, which previous studies on the implementation of complex interventions have also shown (27).

The promotion of participation is another central aspect of the model. The changes in traditional ward structures and conventional treatment processes in respect to consistent transparency and patient participation enables the patient to engage in thought processes and suggestions of the medical team. Ward rounds and treatment planning efforts are held in the presence of the patient. The patient can adjust the treatment procedure including decisions on medications and their dosage. A previous study showed that after the implementation of the Weddinger Modell the number of patients who could not name any treatment goals decreased (14). The mentioned promotion of patient autonomy and self-efficacy is one aspect that is related to a lower level of experienced coercion (18). It requires a Recovery-oriented attitude from the ward staff to enable a person-centered approach in accompanying the patient.

This approach and these above-mentioned central attitudes are maintained and even emphasized in the context of involuntary commitment and involuntary medication. If a patient is at acute risk due to a mental illness, the recommendation for medication is nevertheless justified accordingly. As those affected by forced medication mostly suffer from the impression that they are not adequately informed about the treatment and involved in it (25), decisions for forced medication are explained and discussed with the patient in a way that promotes the patient's participation. It is clearly communicated that the medication is given because the patient currently poses a risk to himself or others.

Limitations of the findings presented affect mainly three points.

Firstly, the retrospective study design allows a comprehensive examination of patient data and a statement on the research question, but it must be taken into account that this design lacks benefits of randomization of study groups and exclusion of confounding factors. A prospective study design would be a useful addition to this research question to establish proof of the hypotheses generated in this study.

Secondly, missing effects in several parameters may be due to the limited sizes of the partial samples of the study groups such as the number of those patients affected by forced medication. Due to the resulting low power, it might have been difficult to obtain statistically significant results. Thus, the present work can only serve as a starting point for future studies with larger samples that are devoted to this research question.

Thirdly, medication parameters were not linked with data regarding length of involuntary admission in this study, which would have enabled a more holistic view.

## Conclusion

Maintaining the promotion of participation, effective multi-professionality, and focus on Recovery even in the context of forced medication certainly belong to the greatest successes of the Weddinger Modell, which is reflected in lower doses of maximum forced medication doses. In combination with our previous findings we conclude that the implementation of the Weddinger Modell led to a reduction of coercion in our in-patient wards (19). Reduction of mechanical coercive measures was not associated with more forced medication or higher doses of psychotropic drugs in our population. Close multi-professional cooperation allows to regularly evaluate therapeutic approaches and crisis situations. Responsibility for therapeutic relationship and (medical) treatment is shared and allows a common assumption of risk. This leads to a rapid relief from the crisis. Recovery-oriented treatment concepts should be apprehensively considered in acute psychiatric care.

## Data Availability Statement

The raw data supporting the conclusions of this article will be made available by the authors, without undue reservation.

## Author Contributions

KC: conceptualization, data collection, analysis, interpretation of the data, writing—original draft, and editing of previous drafts. FB: conceptualization, supervision, interpretation of the data, and critical review. AW: supervision, interpretation of the data, and critical review. LM: conceptualization, study design, supervision, interpretation of the data, and critical review. All authors approved the final version.

## Conflict of Interest

The authors declare that the research was conducted in the absence of any commercial or financial relationships that could be construed as a potential conflict of interest.

## Publisher's Note

All claims expressed in this article are solely those of the authors and do not necessarily represent those of their affiliated organizations, or those of the publisher, the editors and the reviewers. Any product that may be evaluated in this article, or claim that may be made by its manufacturer, is not guaranteed or endorsed by the publisher.
